# Computational fluid–structure interaction analysis of flapping uvula on aerodynamics and pharyngeal vibration in a pediatric airway

**DOI:** 10.1038/s41598-023-28994-2

**Published:** 2023-02-03

**Authors:** Yicheng Chen, Xin Feng, Xie-Qi Shi, Weihua Cai, Biao Li, Yijun Zhao

**Affiliations:** 1 School of Energy Science and Engineering, Harbin Institute of Technology, Harbin, China; 2 Division of Ear, Nose and Throat Surgery, Akerhus University Hospital, Lørenskog, Norway; 3 Institute of Clinical Medicine, University of Oslo, Oslo, Norway; 4 Department of Clinical Dentistry, Section for Oral and Maxillofacial Radiology, University of Bergen, Bergen, Norway; 5 Department of Oral Maxillofacial Radiology, Faculty of Odontology, Malmö University, Malmö, Sweden; 6 School of Energy and Power Engineering, Northeast Electric Power University, Jilin, China

**Keywords:** Computational biophysics, Biological physics

## Abstract

The uvula flapping is one of the most distinctive features of snoring and is critical in affecting airway aerodynamics and vibrations. This study aimed to elucidate the mechanism of pharyngeal vibration and pressure fluctuation due to uvula flapping employing fluid–structure interaction simulations. The followings are the methodology part: we constructed an anatomically accurate pediatric pharynx model and put attention on the oropharynx region where the greatest level of upper airway compliance was reported to occur. The uvula was assumed to be a rigid body with specific flapping frequencies to guarantee proper boundary conditions with as little complexity as possible. The airway tissue was considered to have a uniform thickness. It was found that the flapping frequency had a more significant effect on the airway vibration than the flapping amplitude, as the flapping uvula influenced the pharyngeal aerodynamics by altering the jet flow from the mouth. Breathing only through the mouth could amplify the effect of flapping uvula on aerodynamic changes and result in more significant oropharynx vibration.

## Introduction

Snoring is a common condition in the general population, with a prevalence of 60% and 12.1% in the adult male and pediatric populations^1,2^, respectively. The patients often have recurrent episodes of loud snoring and eventually develop Obstructive sleep apnea (OSA) during sleep^3,4^, which poses noise and health risks. Uvula, as part of the soft palate, can dangle into the airway when sleeping, narrowing the airway and providing a soft tissue that is more prone to vibrate than many other tissues in the airway. If the uvula becomes swollen or irritated, it can worsen the snoring. Invasive uvulopalatopharyngoplasty treatment of the uvula has shown significant results against snoring, demonstrating the critical role of the soft palate and uvula in snoring^5,6^. The large uvula flapping of pediatric patients on inspiration was also confirmed using dynamic magnetic resonance imaging (MRI)^7,8^. Therefore, the uvula motion is believed to be a key factor affecting airway aerodynamics and vibration.

The anatomically accurate 3D fluid–structure interaction (FSI) analysis can be recognized as a promising method to investigate pharyngeal deformation in snoring studies. Some previous studies have performed FSI studies in pharyngeal models without uvula^9–12^. They found a prominent deformation on the oropharynx during inhalation with different breath patterns (etc., the mouth breathing and nose breathing), consistent with Brennick’s medical report^13^. However, as the repeated large vibrations of the uvula can cause snoring by obstructing breathing^14^, the uvula’s aerodynamic role should also be considered for FSI analysis in upper airway studies.

Sun et al.^15^ built finite element models of the uvula from MRI with a rigid upper airway to analyze differences in the airflow field and tissue movement between OSA and health subjects. They pointed out that the OSA patient may have a more significant uvula displacement due to their airways’ remarkably escalating pressure and velocity. In the studies by Zhu^16^ and Wang^17^, passive movement of the human uvulas was studied assuming a laminar and a transitional airflow regime. They found that the displacement of the uvula was caused predominantly by force exerted by static pressure on the tissues’ boundary. Nevertheless, only nose breathing was performed in their studies, and no effects on the airflow downstream were found due to the small displacement of the uvula. Pirnar et al.^18^ built 3D upper airways with surrounding soft tissues to analyze uvula movement. They found a significant uvula flutter during the expiratory phase of breathing which has a similar frequency to the snoring sound reported in other literature^19^. Wang et al.^20^ studied a rigid pharyngeal model with a flapping uvula using Immersed Boundary Method and Direct Numerical Simulation. Their simulations showed a strong relationship between airway pressure and pharyngeal wall force fluctuation with flapping frequencies. However, the width of the pharynx had a more significant impact on airflow than uvula flapping frequencies. These contributions have employed and discussed FSI to simulate uvula flapping on inspiration and expiration so far, but none to the best of our knowledge obtained both the uvula flapping and its effect on pharyngeal airway vibrations downstream.

Additionally, the effect of breathing patterns on snoring sound and spectrum has been reported in medical practice^21^, indicating different physical mechanisms. Some research^9,22^ has studied the role of the nose and nasopharynx in airway deformation. However, it is still poorly understood concerning the airway vibration during uvula flapping under different breathing patterns. The FSI method seems feasible to study airflow characteristics and possible underlying physical mechanisms associated with breathing patterns and has been used to study the corresponding changes in aerodynamics^23^.

The objectives of this study were: (i)—the impact of uvula flapping frequencies and amplitudes on airway flow field characteristics and oropharynx vibration. (ii)—the effect of breathing patterns on pharyngeal aerodynamics and oropharynx vibration, including the mouth, nose, and mouth-nose breathing.

## Results

### Effect of flapping frequencies on airflow field

**Figure 1:**
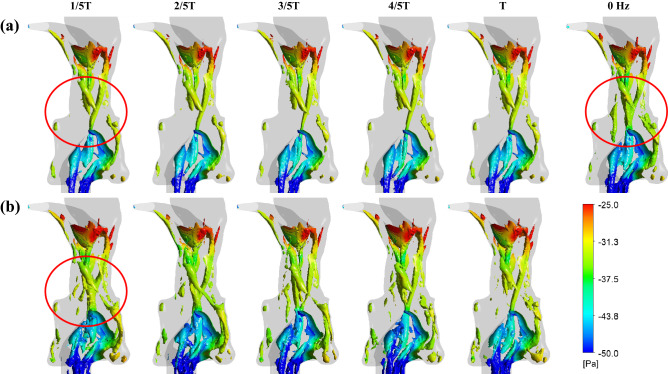
Comparison of airway vortex structures within a uvula flapping period (T) under all flapping frequencies, including 0 Hz (stationary airway), (**a**) 20 Hz, and (**b**) 40 Hz. Three-dimensional wake topologies are visualized using the Liutex-criteria (vortex absolute strength = 500). The iso-surfaces are colored by local pressure.

Figure 1 compares the instantaneous vortex structures in the pharyngeal airways (Liutex-criteria, vortex absolute strength = 500) of three flapping frequencies (*ω*_*r*_, *ω*_*r*1_ = 0 Hz, *ω*_*r*2_ = 20 Hz, *ω*_*r*3_ = 40 Hz) within a flapping period. The air flows through the stenosis velopharynx and flapping uvula, inducing significant vortices over the period at all *ω*_*r*_. The uvula vortex and velopharynx vortex merge and are transported downstream to the narrowed oropharynx, causing complex vortices in the epiglottis region. These vortices do not change during the simulation of *ω*_*r*1_ but periodically swing at *ω*_*r*2_ and *ω*_*r*3_.

**Figure 2:**
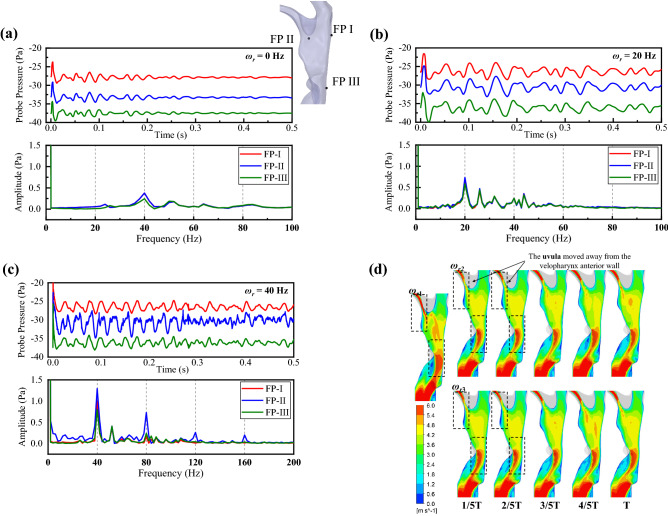
Detailed comparison of the flow field for varying uvula flapping frequencies. (**a**–**c**) Time history signals and corresponding FFT analyses of three pressure probes (FP I, II, and III) at three flapping frequencies. The time-averaged pressure on FP I, II, and III are as follows. *ω*_*r*1_: − 27.865 Pa, − 33.265 Pa, and − 37.508 Pa. *ω*_*r*2_: − 26.317 Pa, − 30.517 Pa, and − 36.108 Pa. *ω*_*r*3_: − 26.537 Pa, − 30.551 Pa, and − 36.166 Pa. (**d**) Airflow velocity contours in the midsagittal plane of the pharyngeal airway.

Figure 2 illustrates the probe pressure signals, the corresponding analyses using the Fast Fourier transform (FFT), and the velocity contours at the midsagittal plane of the pharyngeal airway at different *ω*_*r*_. As shown in Fig. 2a–c, the uvula flapping will slightly increase the time-averaged pressure in all pressure probes (FP I-III) during inspiration and enhance the pressure fluctuation amplitude. In Fig. 2b,c, energy distributions at *ω*_*r*_ dominate the signals, suggesting that the primary frequency of pressure fluctuation inside the pharyngeal airway is the same as *ω*_*r*_. The FFT spectrum of FP II in Fig. 2c exhibits peaks at *ω*_*r*3_ and its harmonics (from the second harmonic to the fourth harmonic), showing a nonlinear aerodynamic response to the uvula flapping (the external forcing) in terms of energy distribution versus frequency. This nonlinear response has little effect on the epiglottis region and posterior velopharyngeal wall because only the second harmonic is found in FP I and III results.

The different *ω*_*r*_ causes comparable velocity predictions in the midsagittal plane of the pharyngeal airway (Fig. 2d). The velocity contours within a flapping period show two high-speed jet flow regions close to the epiglottis and anterior uvula wall. Whereas all *ω*_*r*_ conditions show the high-speed regions to be quite wide and the jet flow close to the anterior uvula wall will affect the velocity close to the uvula. This jet flow was slightly narrow for the *ω*_*r*2_ and *ω*_*r*3_ when the uvula moved away from the velopharyngeal wall (0–1/2 T). Both simulations of *ω*_*r*2_ and *ω*_*r*3_ presented periodic changes downstream of the uvula, which coincided with the vortices swing in Fig. 1. Compared with the stationary airway, the flapping uvula can decrease the airflow velocities at the anterior uvula wall, weaken the pharyngeal jet flows, and periodically change the pressure distribution inside the oropharynx.

### Effect of flapping frequencies on airway deformation

**Figure 3:**
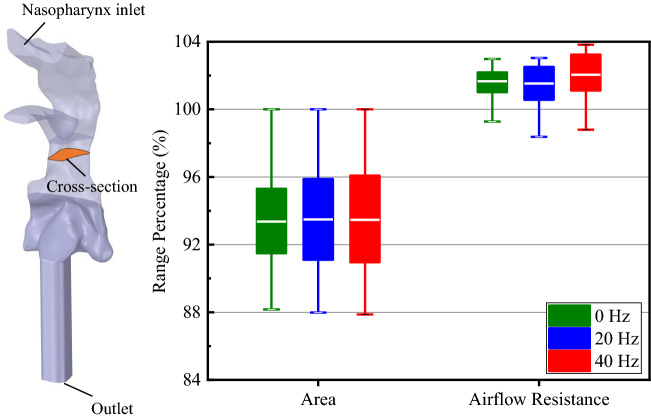
Comparisons of the time-varying cross-sectional area and the pharyngeal airflow resistance at three flapping frequencies. The cross-sectional areas are normalized using the initial value (113.74 mm^2^) to obtain their percentage change during the simulation. The pharyngeal airflow resistance is defined by $R=\Delta P/Q$, where $\Delta P$ is the difference of area-averaged pressure between the nasopharynx inlet and the pharyngeal airway outlet, and $Q$ is the instantaneous airflow rate. The $R$ in this figure is normalized using the initial value of 3.3353 Pa L/min (the airflow resistance of the stationary airway). Data are obtained at each time step during the simulations, and the boxes represent 90% of the data distribution range.

In FSI simulations, the compliant oropharynx structure vibrates due to both material elasticity and the negative pressure caused by inhalation. The cross-sectional area (CSA) fluctuation can be representative of oropharynx vibration. Figure 3 compares the CSA and pharyngeal airflow resistance ($R$) among three *ω*_*r*_. The time-averaged CSA and $R$ are stable in relation to *ω*_*r*_. The fluctuation ranges seems to be higher in the 20 Hz and 40 Hz as compared to the 0 Hz. Therefore, the uvula flapping frequency change does not substantially alter the time-averaged value of oropharynx structure deformation but significantly changed its amplitude of vibration.

**Figure 4:**
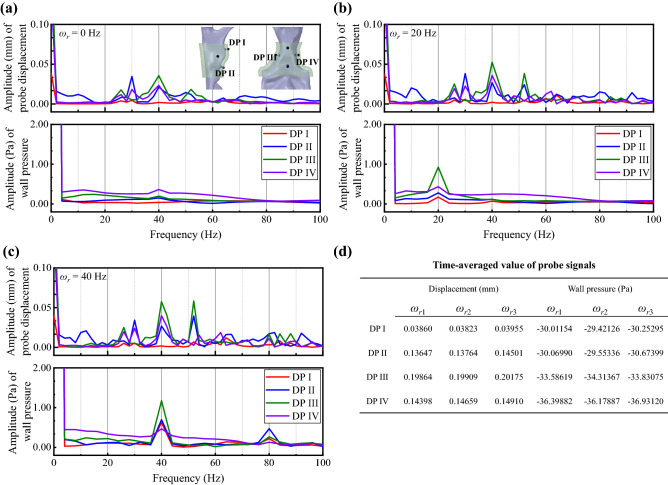
(**a**–**c**) FFT analyses displacement signals from displacement probes (DP I–IV) at three flapping frequencies. Extra one-way FSI simulations were performed to obtain the probe wall pressure close to DPs. The probes for displacement and pressure signals were shown as black points and blue points in (**a**), respectively. The displacement signals were obtained every 5e^−4^ s, and the wall pressure signals were obtained every 1e^−5^ s, which both have sufficient precision over the frequency range of concern. (**d**) The time-averaged value of probe displacement and probe pressure.

A more detailed study of oropharynx deformation compares FFT spectrums of time history signals in displacement probes (DP I–IV) with the corresponding wall pressure FFT spectrums (Fig. 4a–c). Complex wall pressure and displacement responses to the uvula flapping are found concerning energy distribution and frequency. At *ω*_*r*1_ = 0 Hz, the wall pressure signal shows no primary frequency, so there is no periodic external force in the oropharynx structure. There are inborn vibrations around 30 Hz, 40 Hz, and 52 Hz (Fig. 4a). At *ω*_*r*2_ = 20 Hz, the wall pressure shows a linear response to the uvula flapping as it fluctuates at the same frequency with *ω*_*r*_; however, the probe displacements do not show significant responses at the corresponding frequency as no high-energy component around 20 Hz but the energy components around 30 Hz, 40 Hz, and 52 Hz increase (Fig. 4b). Nonlinear response of the wall pressure is found at *ω*_*r*3_ = 40 Hz as the signals exhibited energy distribution at *ω*_*r*_ (*ω*_*r*_ ≠ 0) and its second harmonics that together dominate the signal (Fig. 4c). The energy distributions of probe displacements in Fig. 4c are similar but with higher amplitudes to that of *ω*_*r*2_. The time-averaged values of probe displacement and probe pressure are shown in Fig. 4d.

**Figure 5:**
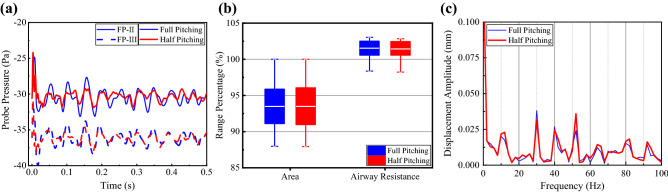
Comparison of the aerodynamic behavior and airway vibration of full flapping amplitude and half amplitude (*ω*_*r*_ = 20 Hz). (**a**) Probe wall pressure of FP II and III. (**b**) The range of CSA and airflow resistance. (**c**) FFT spectrums of probe displacement on DP II.

The reduced uvula flapping amplitude will reduce the external force. However, the half amplitude (*θ*_*r* max_ = 7.5°) only slightly reduces the pressure fluctuation and CSA fluctuation (Fig. 5). Yet, the ranges in Fig. 5b and FFT spectrums in Fig. 5c do not change significantly.

Overall, the uvula flapping frequency is more critical than the flapping amplitude in determining the amplitudes of airway pressure fluctuations and pharyngeal airway deformation. The uvula flapping can significantly alter the wall pressure fluctuations and oropharynx vibration. Nevertheless, the energy components of oropharynx vibrations are not entirely at the same frequencies as the wall pressure fluctuations, suggesting that the pressure fluctuations due to periodic uvula flapping are not the only factor that dominated airway vibration during inhalation.

### Effect of breathing patterns on airway aerodynamic and deformation

**Figure 6:**
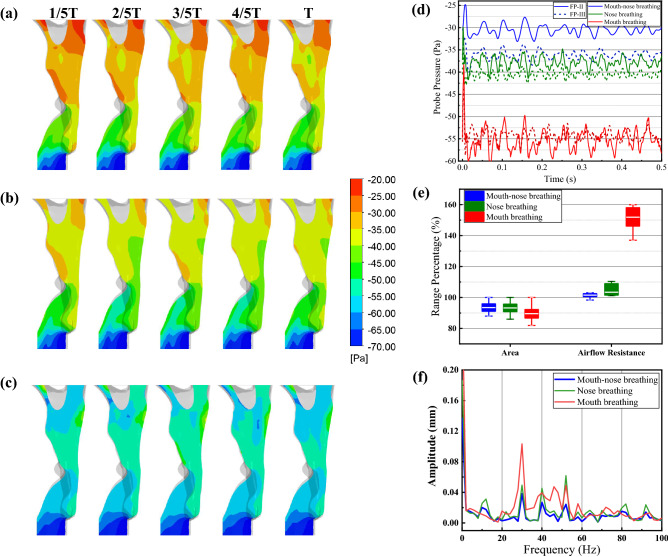
Comparison of aerodynamics and oropharynx vibration characteristics in three breathing patterns. (**a**–**c**) Pressure contours in the pharyngeal midsagittal plane within a flapping period (*ω*_*r*_ = 20 Hz). (**a**) Mouth-nose breathing, (**b**) Nose breathing, (**c**) Mouth breathing. (**d**) Time histories signals of two pressure probes (FP II and III) in three breathing patterns. The time-averaged pressure difference of probes (P_II_–P_III_) were 5.5913, 2.7408, and − 1.0406 Pa of mouth-nose, nose, and mouth breathing, respectively. (**e**) Ranges of CSA fluctuation and airflow resistance. (**f**) FFT analysis of probe displacement on DP IV.

Figure 6 shows the pressure contours, probe pressure signals, CSA and airflow resistance ranges, and FFT analysis of probe deformation signals in three breathing patterns. In the pharyngeal airway, relatively high-pressure regions close to the wall represent a relatively small local cross-wall pressure difference compares to the outer pharyngeal wall pressure (0 Pa). Therefore, the pharyngeal structure deformation will be retarded here. The pressure distribution contours in Fig. 6a confirm relatively high-pressure regions close to the tongue base in mouth-nose breathing pattern. These high-pressure regions disappear when the uvula moves close to the anterior velopharynx wall (from 1/2T to T), which increases the cross-wall pressure difference between the inner and outer walls of the oropharynx structure. In nose breathing pattern, the removal of mouth inflow increases airflow pressure drop inside of pharynx (Fig. 6b). There still exist relatively high-pressure regions close to the tongue base. Thus the high-pressure regions are believed to be a result of the recurrent flow of the nasopharynx airflow. In mouth breathing pattern, the airflow comes through the ’Venturi shape’ constructed by the anterior uvula wall and the velopharyngeal wall with a high-pressure drop (Fig. 6c). The mouth inflow eliminates the high-pressure region when it dominates the pressure distributions in mouth breathing pattern and causes much more pressure drop of airflow than in the other two breathing patterns. The motion of the uvula close to or away from the anterior velopharynx wall has altered the pressure drop of mouth inflow, therefore dominating the pressure distribution inside the oropharynx. This phenomenon is insignificant in mouth-nose breathing pattern (Fig. 6a). Figure 6d compares the probe pressure signals from FP II and III in three breathing patterns. Consistent with the results above, the probe pressure in mouth breathing pattern is much lower than that in the other breathing patterns. In mouth-nose breathing pattern, the probe pressure is the highest but with the largest time-averaged pressure difference between the two probes among the three breathing patterns, which is believed to be a result of the muddying of the nasal and mouth inflows.

Figure 6e shows the ranges of CSA fluctuation and airflow resistance at three breathing patterns. All the ranges of CSA fluctuation are consistent with the same *ω*_*r*_, but the varying breathing patterns cause different time-averaged values. The nose or mouth breathing pattern results in different time-averaged airflow resistance and higher fluctuations ranges compared to the mouth-nose breathing. Given the previously demonstrated effect of uvula flapping on pharyngeal airflow resistance (Fig. 3), this result suggests that the impact of uvula flapping on pharyngeal aerodynamics will be more significant in only mouth or nose breathing pattern than mouth-nose breathing pattern.

Figure 6f of DP II shows the effect of breathing patterns on frequency features of tongue base deformation. FFT spectrums of all conditions exhibit peaks at the same frequencies of 30 Hz, 40 Hz, and 52 Hz, which dominate the signal energy. Airway vibrations are amplified and enhanced at the external forcing (here is uvula flapping) and mouth breathing in terms of the energy distribution versus frequency (Fig. 6f). Overall, the Venturi shape of the mouth inlet in the pharyngeal model, dependent on the small distance between tongue and palate, will result in a jet flow in the oropharynx and dominate the aerodynamics of the pharyngeal airway. Moreover, compared to the other breathing patterns, airway aerodynamics and deformation will be much more sensitive to uvula flapping in mouth breathing pattern.

## Discussion

This study’s reconstructed FSI airway model allowed us to define the uvula flapping characteristics and capture the airflow and airway structure changes. Therefore, the aerodynamic and vibration behaviors in the pharyngeal airway can be studied. This FSI study demonstrates the effect of uvula flapping on aerodynamic and airway deformations in the absence of neurological control and gravity, which has not been reported before. The use of an FSI solver verified in vitro and the anatomically accurate model has promoted the credibility of this study. The pressure boundary setting of the nasopharynx inlet and mouth inlet allowed us to control the airflow rate ratio of mouth and nose inflow during mouth-nose breathing, thus approximating the effect of nasal resistance^24^. The uvula flutter, as the main attribute of snoring, is introduced in our study with specific frequencies based on Osborne’s report^19^. The results of this study will be helpful to better understand airway vibration during snoring from an FSI perspective.

Many factors, that can alter pharyngeal aerodynamics and oropharyngeal deformation, such as uvula flapping properties, breathing patterns, and the airway morphologies of patients, should be considered when studying the FSI of the human upper airway. The current results suggest that uvula flapping frequency rather than amplitude primarily determines the pressure fluctuation and airway vibration within the compliant oropharynx. Likewise, rich airflow characteristics due to uvula flapping are discussed in this study and, to some degree, clarify the mechanism of uvula flutter changing airway aerodynamics. As the Reynold number and flapping frequencies here are smaller than those in Wang’s study^20^, the airway vortices can be continuous and merged periodically. During uvula flapping, the dynamic uvula has periodically blocked the inflow from the mouth and nasopharynx, allowing each to take turns dominating the pressure properties inside the oropharynx at certain times. FSI study without mouth inflow has shown that the ideal excluding of the nasal cavity^9^ will improve the negative pressure inside the oropharynx. However, in physiological condition, the airflow inside the nasal cavity can only be ignoring when the airflow is prevented from getting into the nasal cavity but go through the mouth inlet. Figure 6 illustrates that the mouth breathing worsened negative pressure inside the oropharynx more than nose breathing pattern and mouth-nose breathing pattern due to the patient-specific stenosis between our subject’s soft palate and tongue that caused a dramatic pressure drop in the mouth inlet.

For snoring, the breathing patterns also take into account in this study since the flapping frequency of the uvula can be determined by the cross-wall pressure difference^25–28^. The key results in Fig. 6 indicate that the breathing patterns will amplify or suppress the effect of uvula flapping on the airflow, thereby altering the amplitude of airway vibration. Therefore, it is speculated that nose breathing or keeping nose and mouth completely open may be a better option to avoid severe airway vibration for patients with severe snoring.

One of the potential limitations of this study is the “collapsible homogeneous channel” description of our pharyngeal airway model. The soft tissues of the oropharyngeal structure are reconstructed with uniform thickness and homogeneous material properties with linear elastic. This is different from the real pharyngeal, which is surrounded by separated tissues, leading to the loss of specific deformation detail. Another limitation of this study is that the assuming uvula flapping frequencies and inhale flow rate are not patient-specified. In addition, the current pilot study on one case cannot represent all possible influences due to anatomical variations in the upper airway. Further investigation on the accuracy and reliability of FSI simulation involving more cases is necessary.

## Conclusion

A pharyngeal airway model was constructed to study the interaction among uvula flapping, oropharynx airway structure, and inhaled airflow using computational FSI simulation. Our simulation results based on one pediatric airway demonstrate that the flapping frequencies seem to have a more significant effect on airway vibration than flapping amplitude. Further explanation in terms of aerodynamics is that the flapping uvula influences the aerodynamic behaviors inside the oropharynx by controlling the jet flow through the mouth cavity. In extreme cases, such as breathing only through mouth or nose, the influence of uvula flapping will be amplified or suppressed during inspiration.

## Material and method

### Object and 3D model reconstruction

**Figure 7:**
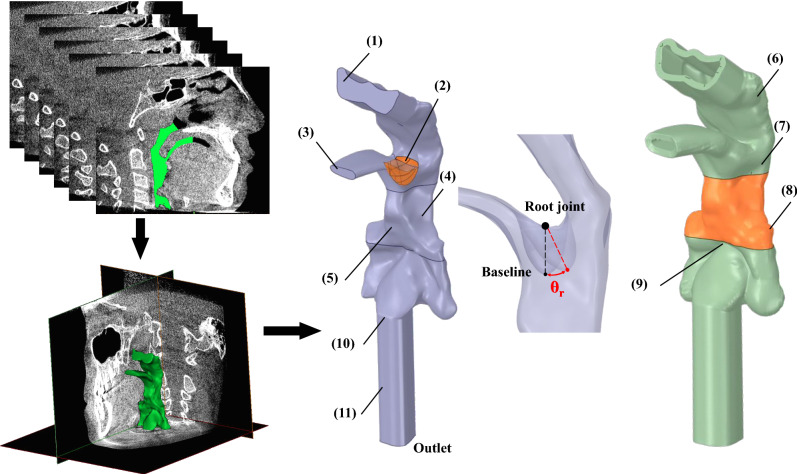
Reconstruction procedure of the fluid region and pharyngeal structure with identified boundary regions. (1) Inlet of nasopharynx, (2) uvula, (3) inlet of oral cavity, (4) tonsil surface, (5) surface of the tongue base, (6) structure of nasopharynx, (7) structure of velopharynx, (8) structure of oropharynx (deformable), (9) epiglottis, (10) bottom of pharyngeal airway, (11) extension.

The object of this study was a 13-year-old boy who underwent a Cone-Beam CT (CBCT) scan at the Department of Orthodontics (Stomatological hospital, Dalian, China). The scans were retrospectively collected and used to reconstruct a 3D model of the upper airway (3D eXam; KaVo, Biberach an der Riss, Germany). The recorded scanning parameters were 120 kV and 5 mA, with a scanning time of 14.7 s. Voxel size was 0.2 mm, and each layer was scanned at a 0.2 mm interval, with 14-bit pixel depth and 13 × 17 cm field of view. The CBCT scan was exported in digital imaging and communications in medicine format for further analysis. The airway boundary was defined using a grayscale threshold from − 1024 to − 800 (approximately − 1000 Hounsfield units for air region) in Mimics 23.0 (Materialise, Belgium) that acquired a 3D segmented UA surface model, including nasopharynx, velopharynx, and soft palate within uvula, part of the oral cavity, oropharynx, and laryngopharynx (Fig. 7). In this study, the inlets (nasal choana and oral cavity) and outlet (base of the epiglottis) were elongated to 10 mm, 2 mm, and 40 mm, respectively^29^. This surface model was filled as a volume model for the fluid domains and a 2 mm thick airway structure for the solid domain^12^.

### FSI governing equations

The estimated range of Reynolds numbers (Re) was between 930 to 2420 in pediatric airway^30,31^, indicating the pharyngeal airflow to be laminar or transitional. In this study, the ANSYS Fluent 14.5 was applied to solve the fluid governing equations of the Low-Reynold-Number SST *k-ω* model, and the ANSYS Mechanical 14.5 was applied to solve the pharyngeal structural equations. The fluid–solid interface can be two-way coupled using the local stress tensor (${\sigma }_{ij}^{s}$ and ${\sigma }_{ij}^{f}$) and displacement data (*D*_*i*_). This FSI method was also further validated using in vitro experiments, testing its reliability for predicting structure displacements in the same pharyngeal model in this study (“Method verification in vitro” section).

The continuity and momentum equations of incompressible airflow in terms of Reynolds averaging are given by Eqs. (2) and (3):

1 $$\begin{array}{*{20}c} {u_{i} = \overline{u}_{i} + u_{i}^{\prime } } \\ \end{array}$$

2 $$\begin{array}{*{20}c} {\frac{\partial }{{\partial x_{i} }}\left( {\rho \overline{u}_{i} } \right) = 0} \\ \end{array}$$

3 $$\begin{array}{*{20}c} {\rho \frac{{\partial \overline{u}_{i} }}{\partial t} + \rho \overline{u}_{i} \frac{\partial }{{\partial x_{i} }}\left( {\overline{u}_{i} } \right) = - \frac{\partial P}{{\partial x_{i} }} + \frac{\partial }{{\partial x_{j} }}\left[ {\mu \left( {\frac{{\partial \overline{u}_{j} }}{{\partial x_{i} }} + \frac{{\partial \overline{u}_{i} }}{{\partial x_{j} }} - \frac{2}{3}\delta_{ij} \frac{{\partial \overline{u}_{j} }}{{\partial x_{i} }}} \right)} \right] + \frac{\partial }{{\partial x_{i} }}\left( { - \rho \overline{{u_{i}^{\prime } u_{j}^{\prime } }} } \right)} \\ \end{array}$$

where ${\overline{u} }_{\text{i}}$ and $u_{i}^{\prime }$ are the mean and fluctuating velocity components, *ρ* is the fluid density, *p* is the fluid pressure, *μ* is the dynamic viscosity, *i* and *j* are the Cartesian coordinates. Additional terms named Reynold stresses ($- \rho \overline{{u_{i}^{\prime } u_{j}^{\prime } }}$) now appear that represent the effects of turbulence.

The air in this study is regarded as a Newtonian fluid. Its flow behavior will raise viscous stresses, which are linearly correlated to the local strain rate over time, at every point. Therefore, stresses are proportional to the rate of change of the fluid’s velocity vector. The Cauchy stress tensor in a Newtonian fluid field is:

4 $$\begin{array}{*{20}c} {{\varvec{\sigma}}_{ij}^{{\varvec{f}}} = - pI + \mu \left( {\left( {\nabla \vec{u} + \left( {\nabla \vec{u}} \right)^{T} } \right) - \frac{2}{3}\nabla \cdot \vec{u}{\varvec{I}}} \right)} \\ \end{array} { }$$

where ${\varvec{I}}$ is the unit tensor, $\overrightarrow{u}$ is the fluid velocity vector. The fluid Cauchy stress tensor will be transferred and mapped on the fluid–solid interface, and continuous forces originating from the fluid will be assigned as the solid boundary conditions.

For the solid body surrounded by fluid, its deformation tensor can be calculated as follow:

5 $$\begin{array}{*{20}c} {{\varvec{\varepsilon}}_{ij} = \frac{1}{2}\left( {\user2{\nabla }\vec{x} + \left( {\user2{\nabla }\vec{x}} \right)^{T} } \right)} \\ \end{array}$$

here ${\varvec{x}}$ is the displacement vector defined on the solid body, which results from the forces and displacements imposed on the fluid–solid interface. The structural material constitutive equation of linear material is defined as

6 $$\begin{array}{*{20}c} {{\varvec{\sigma}}_{{{\varvec{ij}}}}^{{\varvec{s}}} = \frac{E\nu }{{\left( {1 + \nu } \right)\left( {1 - 2\nu } \right)}}tr\left( {{\varvec{\varepsilon}}_{{{\varvec{ij}}}} } \right)I + \frac{E}{{\left( {1 + \nu } \right)}}{\varvec{\varepsilon}}_{{{\varvec{ij}}}} } \\ \end{array}$$

where *E* is Young’s modulus, $\nu$ is the Poisson’s ratio, and ${{\varvec{\sigma}}}_{\text{ij}}^{{\varvec{s}}}$ is the stress tensor. The ${{\varvec{\sigma}}}_{\text{ij}}^{{\varvec{s}}}$ and ${{\varvec{\sigma}}}_{\text{ij}}^{{\varvec{f}}}$ are equal at the fluid–solid interface. For the structure, the momentum equation of the solid is:

7 $$\begin{array}{*{20}c} {\nabla \cdot {\varvec{\sigma}}_{{{\varvec{ij}}}}^{{\varvec{s}}} + {\varvec{F}}_{{\varvec{i}}} = \rho^{s} \frac{{\partial^{2} {\varvec{D}}_{{\varvec{i}}} }}{{\partial t^{2} }}} \\ \end{array}$$

where *ρ*^*s*^ represents the soft tissue density, ${{\varvec{D}}}_{\text{i}}$ represents structural displacement, and the ${{\varvec{F}}}_{\text{i}}$ represents body force, which excluded gravity and the neuromuscular force in this study. The ${{\varvec{D}}}_{\text{i}}$ will be calculated in the structure solver and transferred to the fluid–solid interface.

### Boundary condition

The inlets and outlet of the pharyngeal airway were set as pressure boundaries with constant values, which made the average airflow rate at the outlet close to 20 L/min. To simulate concurrent mouth-nose inspiration, the inlet pressure of the oral cavity was 0 Pa, and the nasopharynx was − 20 Pa, making about 60% of the air go through the nasopharynx and 40% through the mouth^24^. The outlet pressure was − 70 Pa. According to Wang’s study^20^, set the uvula flapping with a rotation angle *θ*_*r*_ of 15° (Fig. 7) and flapping frequency *ω*_*r*_ controlled by Eq. (8) but considered the uvula as a rigid body to simplify the degree of freedom. The center of rotation is set at the root joint (Fig. 7). As the palatal snoring produced explosive peaks of sound at very low frequency (≈ 20 Hz)^19^, to set the base flapping frequency at 20 Hz in accordance with the medical report^19^ and a previous FSI study of soft palate flutter^18^. Flapping frequencies of 40 Hz and 0 Hz were also tested to study the uvular frequency effects. Two-way FSI simulations were performed for each frequency, and extra one-way FSI simulations were conducted to obtain the static wall pressure of the compliant oropharynx structure.

8 $$\begin{array}{*{20}c} {\theta_{r} \left( {\text{t}} \right) = 15^\circ \times \left( {1 - \cos \left( {2{\uppi }\omega_{r} \times {\text{time}}} \right)} \right)} \\ \end{array} { }$$

The compliant airway structure was confined to the oropharynx regions located downstream of the uvula in this study (Fig. 7). This region was previously reported to be the most prone to deformation^32–34^. The cross-sections at the top and bottom of the compliant oropharynx structure were fixed, with an external pressure of 0 Pa on the outer wall of the structure. The airway tissue was defined as a homogeneous and linear elastic material with Young’s modulus of 7540 Pa, a Poisson’s ratio of 0.49, and a density of 920 kg/m^3^^12,15,18^.

The time step was defined as 10^–5^ s, and each simulation contained 0.5 s, which was more than 10 periods of uvula flapping. Every step converged when the residual was smaller than 10^–3^. The FSI interface was set as no-slip wall boundary.

Three pressure probes (FP I–II) were distributed in the pharynx airway to monitor the wall pressure change, including a posterior wall of the velopharynx, downstream of the uvula, and downstream of the oropharynx (Fig. 2a). The airway cross-sectional area (CSA) detection plane was set at the top of the tonsils, where the narrowest region of the oropharynx was (Fig. 3). Four displacement probes (DP I–IV) with corresponding wall pressure probes were distributed on the outer wall of the oropharynx structure (Fig. 4).

This study presented and compared the results from FSI simulations, including the vortex structures, probe signal analysis, airway CSA reductions, probe displacements, and pharyngeal airflow resistance. The 3-D vortex structures were visualized using iso-surfaces defined by vortex absolute strength^35^ using Liutex-based vortex identification and colored by pressure in Section “Effect of flapping frequencies on airflow field”. Here the absolute vortex strength represented twice the rotational angular velocity of the rigid rotational motion part of the local fluid. The pharyngeal airflow resistance was defined as the pressure difference (70 Pa) ratio to the instantaneous airflow rate. Fast Fourier transforms (FFT) analysis was also performed on the probe displacements signal to study the frequency features of airway deformation.

### Grid convergence study

**Figure 8:**
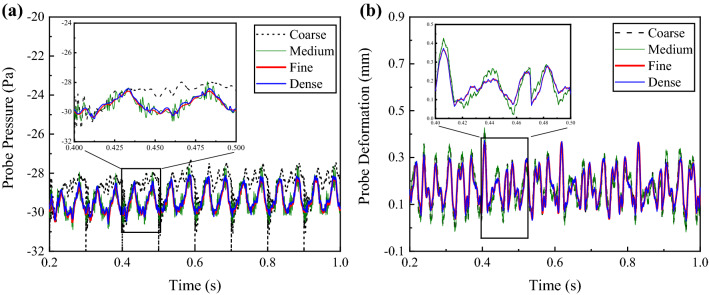
Grids independent study for the instantaneous (**a**) wall pressure and (**b**) probe displacement of structure, respectively. The grid number of the coarse, medium, fine, and dense fluid meshes are 1.31, 2.73, 4.53, and 11.16 million. The grid number of the structure meshes are 0.04, 0.12, 0.2, and 0.46 million.

The grid convergence study was performed to determine the most efficient mesh size for this study. The unstructured tetrahedral volume mesh was applied for the fluid and solid domain. Five layers of hexahedral elements attached to the wall of the fluid domain were used to resolve the viscous sub-layer. The set of calculational meshes is in Table 1. As shown in Fig. 8a, four computational fluid meshes with increasing resolution were included in this study and reached a similar result in Computational Fluid Dynamics simulations on FP II. An additional solid grid convergence study on DP II in FSI simulations was also applied (Fig. 8b). These results show that about 4.53 million computational fluid grids and 0.2 million computational solid grids were adequate for the pre-treatment UA structure.

**Table 1:** Setting of calculational meshes in grid independence study. The height of the prism layers was determined by a transition ratio (0.272) of the height of the last prism and a growth rate (1.2).

	Coarse	Medium	Fine	Dense
Volume grid size for fluid (mm)	0.6	0.5	0.45	0.3
Grid number (million)	1.31	2.73	4.53	11.16
Number of prism layers	5	5	5	5
Transition ratio	0.272	0.272	0.272	0.272
Growth rate	1.2	1.2	1.2	1.2
Total height of prism layers (μm)	585.68	488.03	439.26	298.67
The initial height of prism layers (μm)	78.70	65.58	59.03	39.35
Volume grid size for structure (mm)	0.6	0.5	0.45	0.3
Corresponding Grid number (million)	0.04	0.12	0.2	0.46

### Method verification in vitro

**Figure 9:**
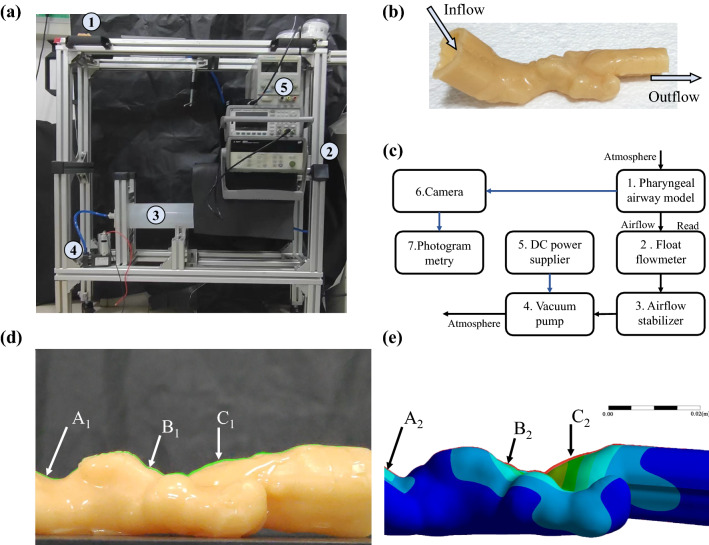
(**a**) Experimental facility for airway deformation measurements in the flexible pharyngeal airway model. (1) The flexible pharyngeal airway model model; (2) Float flowmeter; (3) Airflow stabilizers; (4) Vacuum pump; (5) DC power supplier. (**b**) Flexible UA model. (**c**) Schematic diagram of the experimental facility. The pharyngeal airway deformation of (**d**) Experiment, and (**e**) Simulation. The initial shapes were drawn in green and red. Note that the airway structure was set up as a tube of uniform thickness, so the tongue weight was not considered in this experiment. See the deformation measured in six probes as follow. A1 = 0.390 mm, B1 = 0.354 mm, C1 = 0.552 mm; A2 = 0.2070 mm, B2 = 0.3968 mm, C2 = 0.6080 mm. The error range of the probes of experiments was ± 0.061 mm (± 1 pixel).

An in-vitro airway deformation experimental platform was constructed, and the apparatus is illustrated in Fig. 9. The pharyngeal airway model in Fig. 9b, which excluded mouth^12^, was poured using silicone. The elastic module of soft silicone is 0.94611 MPa (WDW-5Y, Jinan Chuanbai Instrument and Equipment, China), and the Poisson ratio was 0.49. A pump was connected downstream of the airway so the airflow rate could be manually controlled while reading the flow meter. The model was positioned supine on a horizontal table, and its posterior wall was fixed. The critical airflow rate for pharyngeal collapse was determined as 36.4 L/min. The experiments used a high-speed camera (Soni Alpha 7 IV, 3840 * 2160, 60 Hz) to video record instantaneous airway deformation for photogrammetry. The initial shape and the shape at critical airflow rate were both recorded and compared (Fig. 9d,e) using ImageJ.

Approximate boundary conditions were set in the corresponding FSI simulation of 0.1 s, including fixed posterior wall of airway structure, fixed extend, a gravity of − 9.8066 m/s^2^, the critical airflow rate on the outlet, and pressure inlet of 0 Pa. The time-averaged deformation of the simulation is shown in (Fig. 9e) which matches the experimental result with acceptable accuracy and similar distribution.

### Ethical approval

The use of human data had passed ethical review, and all procedures had previously been described^23^. All methods were carried out in accordance with the declaration of research involving human subjects and the regional ethical and scientific guidelines in Dalian, China. The regional ethics review boards approved the study in Dalian, China (Dalian Oral Ethics Committee, DLKQLL201604). The data were retrospectively collected at the Department of Orthodontics (Stomatological hospital, Dalian, China) in 2016. Informed consent was obtained from the patients’ legal guardians.

## Data Availability

The data are available from the corresponding author upon reasonable request.

## List of symbols

*ω*_*r*_: The flapping frequency of the uvula includes 0 Hz (stationary airway), 20 Hz, and 40 Hz
*θ*_*r*_: The maximum rotation angle of the pitched uvula, which represent the amplitude of the uvula flapping
CSA: The cross-sectional area of the fluid region in the airway model
*R*: Airflow resistance of pharyngeal airway
Re: Reynolds number
FP: The static pressure probes inside the fluid domain
DP: The displacement probes on the outer wall of the oropharynx structure
OSA: Obstructive sleep apnea
FSI: Fluid–structure Interaction
MRI: Magnetic resonance imaging
T: A uvula flapping period
FFT: Fast fourier transform
